# Supplementary material for: Spatiotemporal dynamics of homologous recombination repair at single collapsed replication forks
Source: Nat Commun. 2018 Sep 24;9:3882. doi: 10.1038/s41467-018-06435-3 (PMC6155164; doi:10.1038/s41467-018-06435-3)
Supplement: Supplementary file 1 — Supplementary Information [file 41467_2018_6435_MOESM1_ESM.pdf]

Supplementary Information

Spatiotemporal Dynamics of Homologous Recombination Repair at Single Collapsed Replication Forks

Donna R. Whelan, Wei Ting C. Lee, Yandong Yin, Dylan M. Ofri, Keria Bermudez-Hernandez, Sarah Keegan, David Fenyo & Eli Rothenberg*.

Contents:

Supplementary Figures 1-7
Supplementary Tables 1-4
Supplementary References

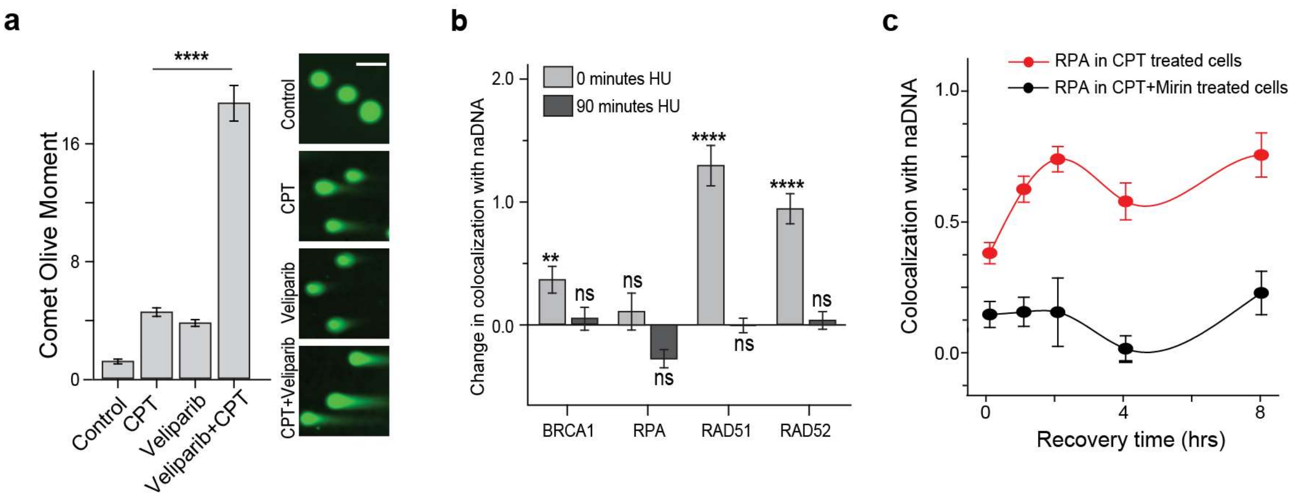

Supplementary Fig. 1

Low doses of CPT generate both stalled RFs which are quickly repaired and seDSBs which persist for several hours and can be monitored by naDNA/RPA foci colocalization

(a) Comet assays of cells damaged with CPT, Veliparib, or both, demonstrating synergistic DSB induction using a combined Veliparib/CPT treatment which confirms the generation of both DSBs and RF stalling in CPT-treated cells. Scale bar, 20 µm. For complete N values see Supplementary Table 3.

(b) Quantification of protein colocalization with naDNA RF foci at unbroken hydroxyurea-stressed RFs immediately following 4 hours of HU treatment confirming association of BRCA1, RAD51, and RAD52 at stalled RFs. After 90 minutes of recovery from hydroxyurea treatment these marker proteins had returned to control levels demonstrating repair of these stalled species within the 90 minutes of recovery. Values are normalized to control levels. For complete N values see Supplementary Table 1.

(c) Kinetic trace of RPA colocalization with naDNA over 8 hours of recovery from CPT treatment either in the presence of Mirin, an MRE11 nuclease inhibitor (black trace) or a DMSO control (red trace). This demonstrates the CPT induction of DSBs that are repaired via HR which can be monitored by the level of RPA colocalization with naDNA RF foci.

Error bars represent mean±s.e.m. Student's t-test for significance as indicated. $^{ns}p > 0.05$, $^{**}p < 0.01$, $^{****}p < 0.0001$.

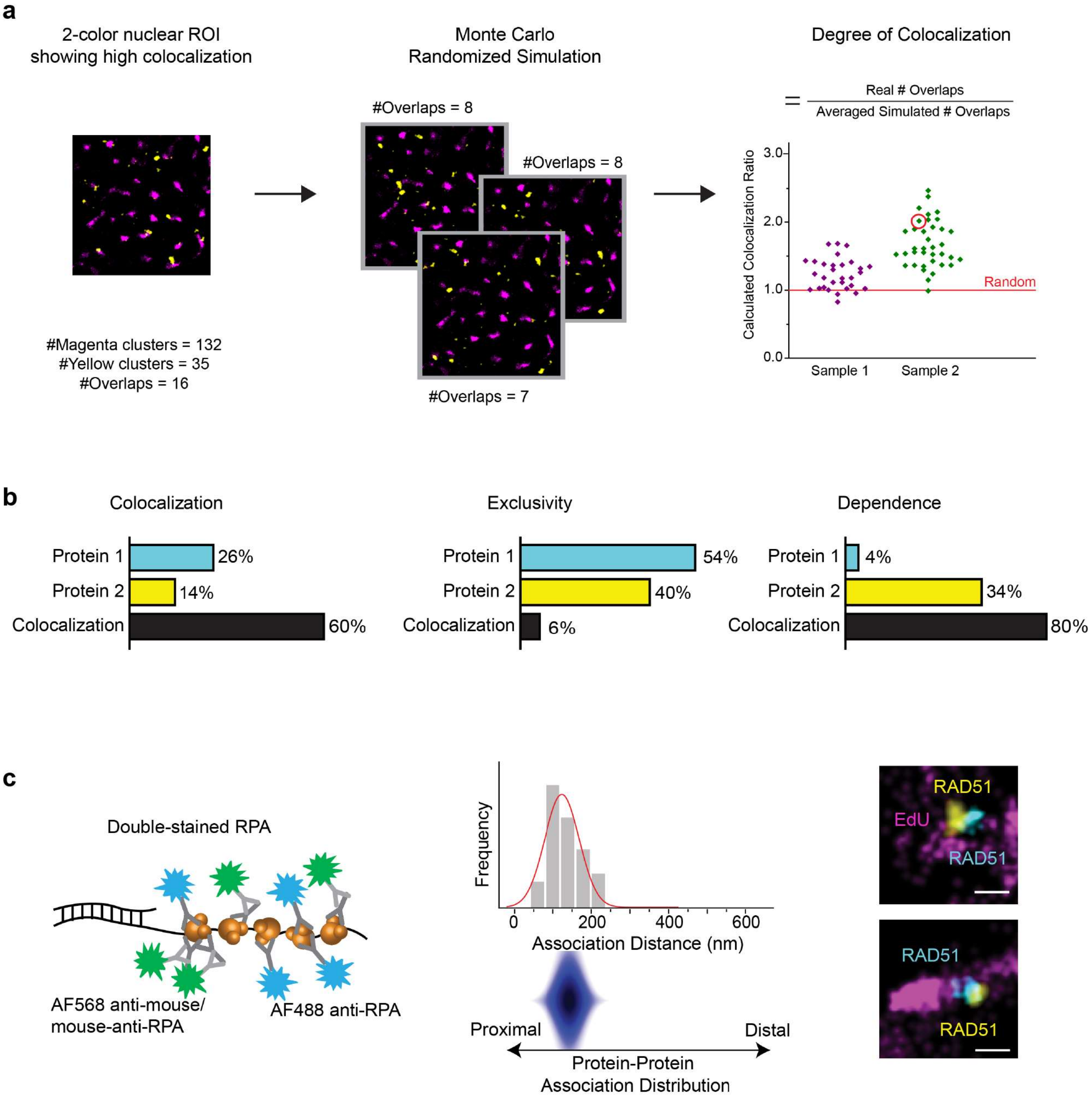

Supplementary Fig. 2

Outlines of the three analytical approaches applied to determine the spatial and temporal behavior proteins at HR repair foci.

(a) Quantification of protein overlap with naDNA was achieved but manually identifying a region of interest and applying an automated threshold to two color channels at a time (magenta and yellow in the example). The clusters of one color within the ROI were then randomly redistributed within the area (yellow) while holding the other colored clusters stationary (magenta). A total of 20 such randomized simulations were generated. Finally, the ratio of overlaps in the real image

to the average number in the simulations was plotted for every cell (for example, the red circled data point from the images shown) allowing for comparison across time points, to control levels, and to the expected random level (=1). The ratioed total number of overlaps per cell sensitively describe the kinetics of proteins expected to interact with DSBs in low numbers, whereas the ratioed area of overlaps better described proteins expected to accumulate, such as RAD51, BRCA2, and RPA.

(b) If proteins were found to colocalize with naDNA after damage to a degree above control levels then this relationship was further assessed by quantifying the percentage of naDNA foci within the sample that were positive for one or both of the proteins under examination. In this way we could discern the predominance of (i) colocalization, (ii) exclusivity, whereby the presence of one protein excluded the second protein from associating with the same foci, and (iii) dependence, whereby the presence of one protein was dependent on the presence of the other.

(c) Pairwise labeling of proteins that yielded a large number of three color foci in which both proteins were associated with the same naDNA were examined to discern whether the proteins were interacting with the repair foci in a proximal or distal arrangement, i.e. whether they were potentially complexed/interacting, versus spatially separated and acting independently. To achieve this, three color foci were cropped and the centers of mass of the two colored foci measured. These distances were then histogrammed and transformed into a 3D protein-protein distribution map by fitting perpendicular Gaussians based on the intensity of the histogram. By double-staining RAD51 nucleofilaments with both Alexa Fluor 488 and 568 we successfully modeled the expected protein-protein distribution map expected of proximal, complexed proteins. Scale bar, 250 nm.

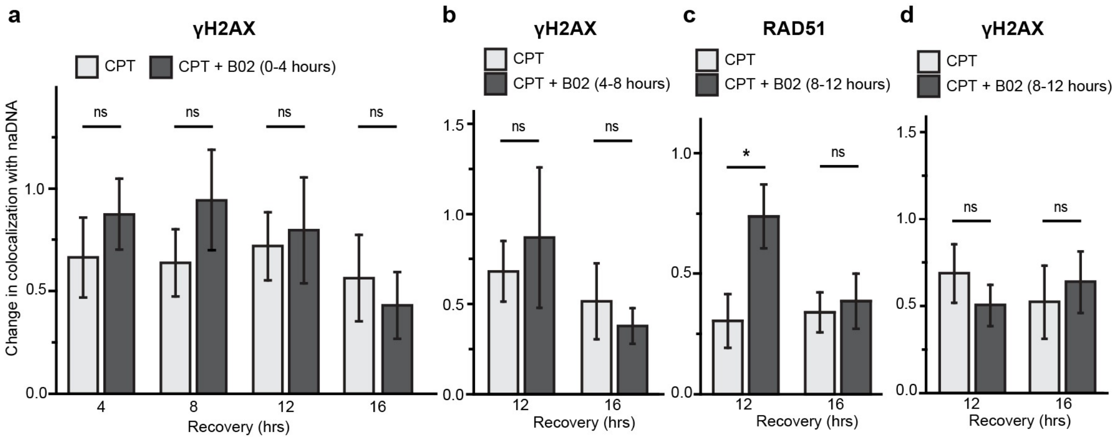

Supplementary Fig. 3

B02 inhibition of RAD51 function does not prolong damage response once it is removed.

(a) Quantification of γH2AX colocalization with naDNA following early (0-4 hours into recovery from CPT) B02 treatment.

(b) Quantification of γH2AX colocalization with naDNA following intermediate (4-8 hours into recovery from CPT) B02 treatment.

(c) Quantification of RAD51 colocalization with naDNA following late (8-12 hours into recovery from CPT) B02 treatment.

(d) Quantification of γH2AX colocalization with naDNA following late (8-12 hours into recovery from CPT) B02 treatment.

For complete N values see Supplementary Table 1. Error bars represent mean±s.e.m. Student's t-test for significance between control and specified conditions. $^{ns}p > 0.05$, $^{*}p < 0.05$.

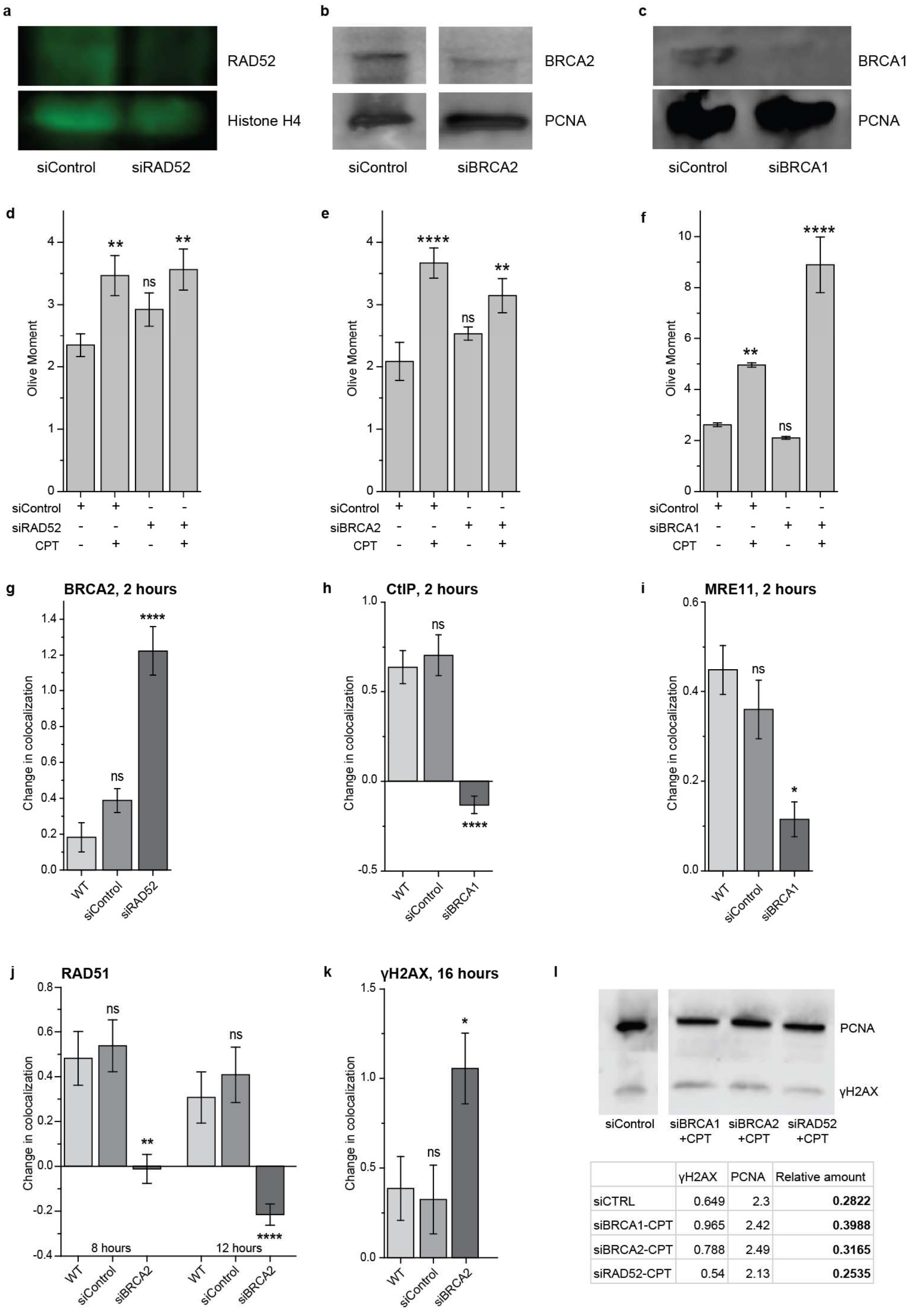

	γH2AX	PCNA	Relative amount
siCTRL	0.649	2.3	**0.2822**
siBRCA1-CPT	0.965	2.42	**0.3988**
siBRCA2-CPT	0.788	2.49	**0.3165**
siRAD52-CPT	0.54	2.13	**0.2535**

Supplementary Fig. 4

Control western blot, comet, and super resolution experiments show minimal impact from siRNA treatments.

(a-c) Western blot confirmation of successful knockdown of RAD52, BRCA2, and BRCA1 in U2OS cells as compared to siLuciferase control treated cells.

(d-f) Comet assays further confirmed small but not statistically significant increases in the number of DSBs generated in siRNA-treated cells without CPT treatment, as well as generation of DSBs upon CPT damage. We further confirmed that siControl treatments did not alter the HR pathway by comparing the colocalization of key HR proteins detected in siControl+CPT cells with both WT+CPT and siRNA+CPT cells. All cells shown were treated with CPT. For complete N values see Supplementary Table 3.

(g) BRCA2 was observed at 2 hours to be very similar in siControl and WT cells (at undamaged levels), but significantly increased in siRAD52 cells. For complete N values see Supplementary Table 1.

(h) CtIP was observed at 2 hours to be similarly elevated above undamaged levels in both WT and siControl cells, as compared to the diminished colocalization detected in siBRCA1 cells. For complete N values see Supplementary Table 1.

(i) Colocalization of MRE11 in siControl and WT cells at 2 hours was detected at elevated levels while slightly diminished in siBRCA1 cells. For complete N values see Supplementary Table 1.

(j) RAD51 was also detected at elevated levels in both siControl and WT cells at both 8 and 12 hours as compared with the low levels detected in siBRCA2 cells. For complete N values see Supplementary Table 1.

(k) γH2A.X overlap was elevated in siBRCA2 cells but close to control levels in both siControl and WT. For complete N values see Supplementary Table 1.

(l) Western blot analysis of γH2A.X persistence 24 hours after release from CPT treatment in siRNA-treated cells confirming increased persistent damage in BRCA1- and BRCA2-depleted cells. Together these results demonstrate the successful siRNA transfection of cells, their susceptibility to CPT damage, and the lack of impact on the HR process in cells siControl treated.

Error bars represent mean±s.e.m. Student's t-test for significance as indicated. $^{ns}p > 0.05$, $^{*}p < 0.05$, $^{**}p < 0.01$, $^{****}p < 0.0001$.

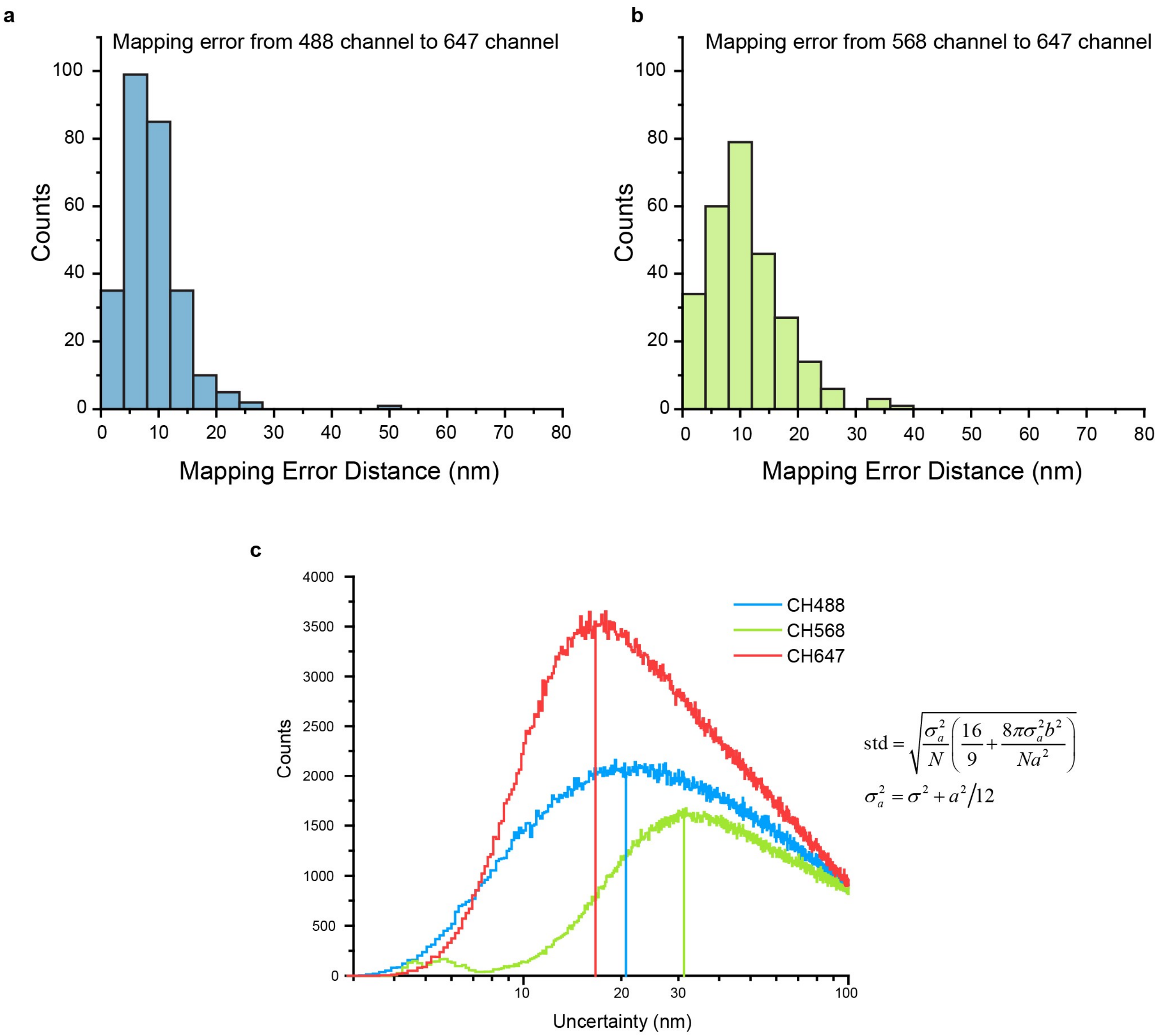

Supplementary Fig. 5

Calculation of mapping and localization errors.

(a-b) To evaluate the mapping errors, we imaged Tetraspeck beads (> 300 beads for one experimental set) in 3 color channels (647, 568, and 488 nm channels), and transformed the beads positions in 488 and 568 channels using the same polynomial functions generated during channel alignment (see Methods), respectively. The Mapping Error was then evaluated by calculating the distance from the transformed beads position to their reference positions in 647 channels. Usually, the mapping error from the 488 channel to the 647 (ref channel) is < 10 nm while the mapping error from 568 channel to the 647 (ref channel) is ~ 10 nm.

(c) The fitting uncertainty (std) was estimated following the formula given in the figure, where s is the fitted sigma of a Gaussian modeled PSF (we use half of the FWHM given by QuickPalm, which is a little bigger than the sigma), and a is the dimension of the pixel size; b stands for the camera background and N denotes the photon number of each blinking event. The maximum of the uncertainty distribution peaks at ~16, 21, 31 nm for the 647, 488, and 568 channels, respectively. The distribution can be fitted into an exponential modified Gaussian distribution, resulting in centers of such Gaussian distributions a bit left-shift from their maximum locations (~9.5, 9.3, 17.7 nm for CH647, CH488, and CH568, respectively).

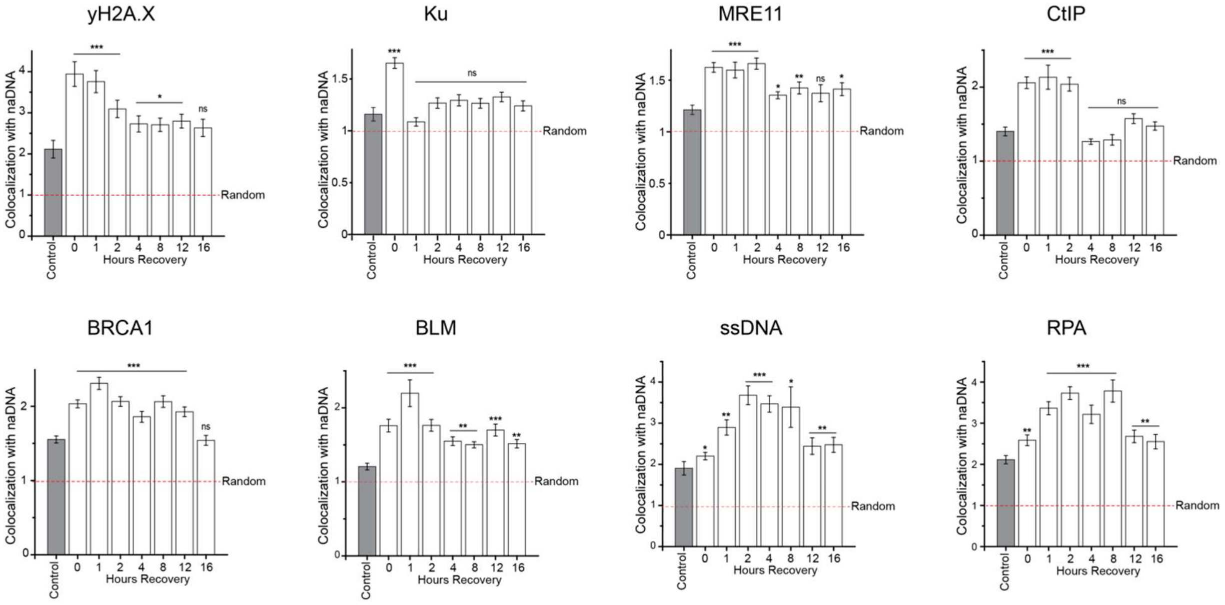

<u>Supplementary Fig. 6</u>

Students t-tests of time course experiments.

To construct the heatmap showing the arrivals, accumulations and departures of proteins at DSB foci, the colocalization of proteins, ssDNA, and the histone modification yH2A.X with naDNA were quantified. Both the degree of colocalization above random and above control levels were considered. Using the Student's two sample t test we have determined the significance of colocalization above control levels, elucidating the detectable kinetics of HRR.

For complete N values see Supplementary Table 1. Error bars represent mean±s.e.m. Student's t-test for significance between damaged and undamaged. $^{ns}p > 0.05$, $*p < 0.05$, $**p < 0.01$, $***p < 0.001$, $****p < 0.0001$.

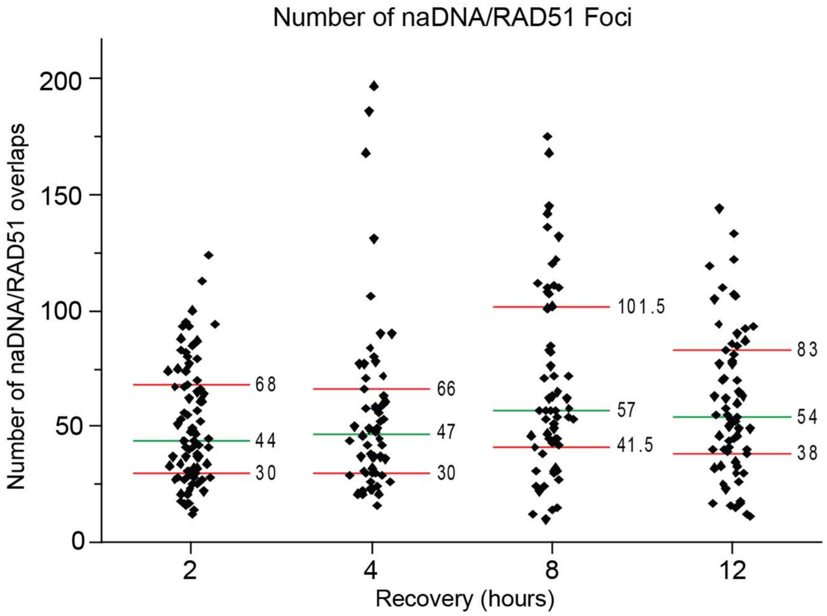

Supplementary Fig. 7

Raw number of naDNA/RAD51 foci show no decrease in detected cluster count due to foci merging upon chromatin reorganization. Cells with less than 10 or more than 200 naDNA/RAD51 overlapping foci were excluded. Red lines indicate 25% and 75% quartiles, green lines indicate the median.

Supplementary Table 1: N values for overlap analyses.

Drug Condition	Time	Species 1	Species 2	N
Control	0	naDNA	yH2A.X	30
CPT Only	0	naDNA	yH2A.X	40
CPT Only	1	naDNA	yH2A.X	36
CPT Only	2	naDNA	yH2A.X	48
CPT Only	4	naDNA	yH2A.X	26
CPT Only	8	naDNA	yH2A.X	37
CPT Only	12	naDNA	yH2A.X	55
CPT Only	16	naDNA	yH2A.X	36
CPT+B02(early)	4	naDNA	yH2A.X	23
CPT+B02(early)	8	naDNA	yH2A.X	20
CPT+B02(early)	12	naDNA	yH2A.X	14
CPT+B02(early)	16	naDNA	yH2A.X	12
CPT+B02(mid)	12	naDNA	yH2A.X	25
CPT+B02(mid)	16	naDNA	yH2A.X	67
CPT+B02(late)	8	naDNA	yH2A.X	18
CPT+B02(late)	12	naDNA	yH2A.X	38
CPT+B02(late)	16	naDNA	yH2A.X	33
CPT+siRAD52	12	naDNA	yH2A.X	21
CPT+siRAD52	16	naDNA	yH2A.X	22
CPT+siBRCA2	12	naDNA	yH2A.X	25
CPT+siBRCA2	16	naDNA	yH2A.X	25
CPT+siControl	16	naDNA	yH2A.X	19
Control	0	naDNA	Ku	19
CPT Only	0	naDNA	Ku	103
CPT Only	1	naDNA	Ku	51
CPT+B02(early)	4	naDNA	Ku	14
CPT+siRAD52	0	naDNA	Ku	47
CPT+siBRCA2	0	naDNA	Ku	25
Control	0	naDNA	MRE11	37
CPT Only	0	naDNA	MRE11	96
CPT Only	1	naDNA	MRE11	48
CPT Only	2	naDNA	MRE11	104
CPT Only	4	naDNA	MRE11	76
CPT Only	8	naDNA	MRE11	37
CPT Only	12	naDNA	MRE11	31
CPT Only	16	naDNA	MRE11	52
CPT+B02(early)	4	naDNA	MRE11	16

Drug Condition	Time	Species 1	Species 2	N
CPT+siBRCA1	0	naDNA	MRE11	18
CPT+siBRCA1	1	naDNA	MRE11	15
CPT+siBRCA1	2	naDNA	MRE11	17
CPT+siRAD52	2	naDNA	MRE11	34
CPT+siBRCA2	2	naDNA	MRE11	36
CPT+siControl	2	naDNA	MRE11	31
Control	0	naDNA	CtIP	35
CPT Only	0	naDNA	CtIP	74
CPT Only	1	naDNA	CtIP	47
CPT Only	2	naDNA	CtIP	74
CPT Only	4	naDNA	CtIP	48
CPT Only	8	naDNA	CtIP	22
CPT Only	12	naDNA	CtIP	43
CPT Only	16	naDNA	CtIP	51
CPT+siBRCA1	0	naDNA	CtIP	74
CPT+siBRCA1	1	naDNA	CtIP	32
CPT+siBRCA1	2	naDNA	CtIP	39
CPT+siControl	2	naDNA	CtIP	23
Control	0	naDNA	BRCA1	46
CPT Only	0	naDNA	BRCA1	81
CPT Only	1	naDNA	BRCA1	59
CPT Only	2	naDNA	BRCA1	68
CPT Only	4	naDNA	BRCA1	52
CPT Only	8	naDNA	BRCA1	36
CPT Only	12	naDNA	BRCA1	39
CPT Only	16	naDNA	BRCA1	47
Control	0	naDNA	BLM	26
CPT Only	0	naDNA	BLM	32
CPT Only	1	naDNA	BLM	47
CPT Only	2	naDNA	BLM	50
CPT Only	4	naDNA	BLM	53
CPT Only	8	naDNA	BLM	64
CPT Only	12	naDNA	BLM	33
CPT Only	16	naDNA	BLM	48

Drug Condition	Time	Species 1	Species 2	N
Control	0	naDNA	BrdU	16
CPT Only	0	naDNA	BrdU	52
CPT Only	1	naDNA	BrdU	62
CPT Only	2	naDNA	BrdU	48
CPT Only	4	naDNA	BrdU	77
CPT Only	8	naDNA	BrdU	33
CPT Only	12	naDNA	BrdU	40
CPT Only	16	naDNA	BrdU	54
Control	0	naDNA	RPA	63
CPT Only	0	naDNA	RPA	58
CPT Only	1	naDNA	RPA	62
CPT Only	2	naDNA	RPA	116
CPT Only	4	naDNA	RPA	68
CPT Only	8	naDNA	RPA	68
CPT Only	12	naDNA	RPA	66
CPT Only	16	naDNA	RPA	58
CPT+B02(early)	4	naDNA	RPA	30
CPT+siBRCA1	0	naDNA	RPA	29
CPT+siBRCA1	1	naDNA	RPA	36
CPT+siBRCA1	2	naDNA	RPA	16
CPT+siBRCA1	4	naDNA	RPA	29
CPT+siRAD52	2	naDNA	RPA	45
CPT+siBRCA2	2	naDNA	RPA	30
Control	0	naDNA	BRCA2	30
CPT Only	0	naDNA	BRCA2	42
CPT Only	1	naDNA	BRCA2	44
CPT Only	2	naDNA	BRCA2	44
CPT Only	4	naDNA	BRCA2	50
CPT Only	8	naDNA	BRCA2	30
CPT Only	12	naDNA	BRCA2	43
CPT Only	16	naDNA	BRCA2	32
CPT+B02(mid)	12	naDNA	BRCA2	24
CPT+siRAD52	0	naDNA	BRCA2	51
CPT+siRAD52	1	naDNA	BRCA2	35
CPT+siRAD52	2	naDNA	BRCA2	38
CPT+siRAD52	4	naDNA	BRCA2	27
CPT+siRAD52	8	naDNA	BRCA2	38
CPT+siRAD52	12	naDNA	BRCA2	47

Drug Condition	Time	Species 1	Species 2	N
CPT+siBRCA1	2	naDNA	BRCA2	36
CPT+siBRCA1	4	naDNA	BRCA2	35
CPT+siBRCA1	8	naDNA	BRCA2	40
CPT+siBRCA1	12	naDNA	BRCA2	28
CPT+siBRCA1	16	naDNA	BRCA2	38
CPT+siRAD52/BRCA1	0	naDNA	BRCA2	28
CPT+siRAD52/BRCA1	2	naDNA	BRCA2	31
CPT+siControl	2	naDNA	BRCA2	32
Control	0	naDNA	RAD52	53
CPT Only	0	naDNA	RAD52	47
CPT Only	1	naDNA	RAD52	63
CPT Only	2	naDNA	RAD52	50
CPT Only	4	naDNA	RAD52	46
CPT Only	8	naDNA	RAD52	40
CPT Only	12	naDNA	RAD52	20
CPT Only	16	naDNA	RAD52	13
CPT+siBRCA2	4	naDNA	RAD52	25
CPT+siBRCA2	8	naDNA	RAD52	30
CPT+siBRCA2	12	naDNA	RAD52	26
Control	0	naDNA	RAD51	67
CPT Only	0	naDNA	RAD51	57
CPT Only	1	naDNA	RAD51	49
CPT Only	2	naDNA	RAD51	50
CPT Only	4		RAD51	47
CPT Only	8	naDNA	RAD51	53
CPT Only	12	naDNA	RAD51	42
CPT Only	16	naDNA	RAD51	34
CPT+B02(early)	4	naDNA	RAD51	43
CPT+B02(early)	8	naDNA	RAD51	24
CPT+B02(early)	12	naDNA	RAD51	26
CPT+B02(early)	16	naDNA	RAD51	21
CPT+B02(mid)	8	naDNA	RAD51	20
CPT+B02(mid)	12	naDNA	RAD51	40
CPT+B02(mid)	16	naDNA	RAD51	39
CPT+B02(late)	8	naDNA	RAD51	33
CPT+B02(late)	12	naDNA	RAD51	31
CPT+B02(late)	16	naDNA	RAD51	31
CPT+siRAD52	0	naDNA	RAD51	51

Drug Condition	Time	Species 1	Species 2	N
CPT+siRAD52	2	naDNA	RAD51	34
CPT+siRAD52	8	naDNA	RAD51	31
CPT+siRAD52	12	naDNA	RAD51	45
CPT+BRCA2	0	naDNA	RAD51	39
CPT+BRCA2	2	naDNA	RAD51	31
CPT+BRCA2	8	naDNA	RAD51	31
CPT+BRCA2	12	naDNA	RAD51	52
CPT+siBRCA1	0	naDNA	RAD51	32
CPT+siBRCA1	2	naDNA	RAD51	24
CPT+siBRCA1	8	naDNA	RAD51	40
CPT+siBRCA1	12	naDNA	RAD51	28
CPT+siBRCA1	16	naDNA	RAD51	38
CPT+siRAD52/BRCA1	0	naDNA	RAD51	52
CPT+siRAD52/BRCA1	2	naDNA	RAD51	45
CPT+siControl	8	naDNA	RAD51	29
CPT+siControl	12	naDNA	RAD51	22
HU	0	naDNA	RAD51	34
HU	1.5	naDNA	RAD51	39
HU	0	naDNA	RAD52	25
HU	1.5	naDNA	RAD52	31
HU	0	naDNA	BRCA1	30
HU	1.5	naDNA	BRCA1	30
HU	0	naDNA	RPA	34
HU	1.5	naDNA	RPA	39
CPT+Mirin	0	naDNA	RPA	16
CPT+Mirin	1	naDNA	RPA	16
CPT+Mirin	2	naDNA	RPA	19
CPT+Mirin	4	naDNA	RPA	17
CPT+Mirin	8	naDNA	RPA	28

Supplementary Table 2: N values for intrafoci analyses of WT+CPT damaged cells.

Time	Species 1	Species 2	N
2	RAD52	RAD51	112
2	RPA	RAD51	77
4	BRCA2	RAD51	69
8	RPA	RAD51	78
8	BRCA2	RAD51	73

Supplementary Table 3: N values for comet assays.

Cell condition	N
siControl	100
siControl + CPT	103
siRAD52	126
siRAD52+CPT	104
siControl	122
siControl + CPT	159
siBRCA2	131
siBRCA2+CPT	130
siControl	95
siControl + CPT	100
siBRCA1	90
siBRCA1+CPT	64

Supplementary Table 4: Antibody List.

Target	Species/Conjugate	Product Code	Manufacturer	Dilutions	Refs
BLM	mouse monoclonal	SC13584	Santa Cruz	1:500/1:2000	1
BRCA1	mouse monoclonal	SC6954	Santa Cruz	1:500/1:2000	2
BRCA1	mouse monoclonal AF488 conjugated	NB100-598AF488	Novus	1:250	*
BRCA2	rabbit polyclonal	NBP1-88361	Novus	1:500/1:2000	3
BRCA2	mouse monoclonal AF488 conjugated	NB600-445AF488	Novus	1:250	*
BrdU	mouse monoclonal	AB8039	Abcam	1:200/1:500	4
CtIP	mouse monoclonal	SC271339	Santa Cruz	1:500/1:2000	5
MRE11	mouse monoclonal	NB100-473AF488	Novus	1:200	6
MRE11	rabbit polyclonal	NB100-142	Novus	1:500/1:2000	7,8
RAD51	rabbit monoclonal AF488 conjugated	AB196449	Abcam	1:250	*
RAD51	rabbit polyclonal	39194	Active Motif	1:500/1:1000	9
RAD51	mouse monoclonal	GTX70230	Genetex	1:500/1:2000	10
RAD52	rabbit polyclonal	SC8350	Santa Cruz	1:400/1:2000	11
RPA	mouse monoclonal	AB2175	Abcam	1:500/1:2000	12,13
RPA	rabbit monoclonal AF488 conjugated	AB199097	Abcam	1:300	14
yH2A.X	rabbit polyclonal	NB100-384	Novus	1:2000/1:10000	15,16
yH2A.X	mouse monoclonal	05-636	EMD Millipore	1:2000/1:10000	17
	goat-anti-rabbit AF568	A11036	Invitrogen		
	goat-anti-rabbit AF488	A11034	Invitrogen		
	goat-anti-mouse AF568	A11031	Invitrogen		
	goat anti-mouse AF488	A11029	Invitrogen		

* denotes antibodies used which had not been used for IF applications in publications previously. To validate these antibodies they were double-stained alongside validated antibodies for the same target and found to have good colocalization.

Supplementary References

1 Petsalaki, E., Dandoulaki, M., Morrice, N. & Zachos, G. Chk1 protects against chromatin bridges by constitutively phosphorylating BLM serine 502 to inhibit BLM degradation. *J. Cell Sci.* **127**, 3902-3908 (2014).

2 Castella, M. *et al.* FANCI regulates recruitment of the FA core complex at sites of DNA damage independently of FANCD2. *PLoS Genet.* **11**, doi:10.1371/journal.pgen.1005563 (2015).

3 Woditschka, S. *et al.* DNA double-strand break repair genes and oxidative damage in brain metastasis of breast cancer. *JNCI-J. Natl. Cancer I.* **106**, doi:10.1093/jnci/dju145 (2014).

4 Kranz, D., Dohmesen, C. & Dobbelstein, M. BRCA1 and Tip60 determine the cellular response to ultraviolet irradiation through distinct pathways. *J. Cell Biol.* **182**, 197-213 (2008).

5 Huhn, D., Kousholt, A. N., Sorensen, C. S. & Sartori, A. A. miR-19, a component of the oncogenic miR-17 similar to 92 cluster, targets the DNA-end resection factor CtIP. *Oncogene* **34**, 3977-3984 (2015).

6 Uziel, T. *et al.* Requirement of the MRN complex for ATM activation by DNA damage. *EMBO J.* **22**, 5612-5621 (2003).

7 Lee, K. Y. *et al.* MCM8-9 complex promotes resection of double-strand break ends by MRE11-RAD50-NBS1 complex. *Nat. Commun.* **6**, doi:10.1038/ncomms8744 (2015).

8 Gao, M. *et al.* Ago2 facilitates Rad51 recruitment and DNA double-strand break repair by homologous recombination. *Cell Res.* **24**, 532-541 (2014).

9 Bennett, B. T. & Knight, K. L. Cellular localization of human Rad51C and regulation of ubiquitin-mediated proteolysis of Rad51. *J. Cell. Biochem.* **96**, 1095-1109 (2005).

10 Pfaffle, H. N. *et al.* EGFR-activating mutations correlate with a Fanconi Anemia-like cellular phenotype that includes PARP inhibitor sensitivity. *Cancer Res.* **73**, 6254-6263 (2013).

11 Wray, J., Liu, J. M., Nickoloff, J. A. & Shen, Z. Y. Distinct RAD51 associations with RAD52 and BCCIP in response to DNA damage and replication stress. *Cancer Res.* **68**, 2699-2707 (2008).

12 Leung, J. W. *et al.* Nucleosome acidic patch promotes RNF168-and RING1B/BMI1-dependent H2AX and H2A ubiquitination and DNA damage signaling. *PLoS Genet.* **10**, doi:10.1371/journal.pgen.1004178 (2014).

13 Carvalho, S. *et al.* SETD2 is required for DNA double-strand break repair and activation of the p53-mediated checkpoint. *Elife* **3**, doi:10.7554/eLife.02482 (2014).

14 Toledo, L. I. *et al.* ATR prohibits replication catastrophe by preventing global exhaustion of RPA. *Cell* **155**, 1088-1103 (2013).

15 Markova, E. *et al.* DNA repair foci and late apoptosis/necrosis in peripheral blood lymphocytes of breast cancer patients undergoing radiotherapy. *Int. J. Radiat. Biol.* **91**, 934-945 (2015).

16 Francia, S., Cabrini, M., Matti, V., Oldani, A. & di Fagagna, F. D. DICER, DROSHA and DNA damage response RNAs are necessary for the secondary recruitment of DNA damage response factors. *J. Cell Sci.* **129**, 1468-1476 (2016).

17 Eren, M. K., Kilincli, A. & Eren, O. Resveratrol Induced premature senescence is associated with DNA damage mediated SIRT1 and SIRT2 down-regulation. *PLoS One* **10**, doi:10.1371/journal.pone.0124837 (2015).
